# A key heavy metal-binding protein orchestrates plant resistance against a geminivirus

**DOI:** 10.1016/j.fmre.2024.12.005

**Published:** 2024-12-27

**Authors:** Hui Liu, Tao Hu, Fangfang Li, Yaqin Wang, Yuzhen Mei, Xueping Zhou

**Affiliations:** a State Key Laboratory of Rice Biology and Breeding, Institute of Biotechnology, Zhejiang University, Hangzhou 310058, China; b State Key Laboratory for Biology of Plant Diseases and Insect Pests, Institute of Plant Protection, Chinese Academy of Agricultural Sciences, Beijing 100193, China

**Keywords:** Heavy metal-binding proteins, Defense, Geminivirus, Tomato yellow leaf curl China virus, Autophagy

## Abstract

Heavy metal-binding proteins (HMPs) are crucial for heavy metal ion transportation in plants, which is involved in plant nutrient absorption, morphogenesis, and immune responses. However, the molecular mechanism enabling the role of HMPs in plant defense in the presence of heavy metal ions has so far remained elusive. Here we show that NbHMP04 mediates host defenses against the geminivirus tomato yellow leaf curl China virus (TYLCCNV) by enhancing the stability of NbNPR1, and that this action is countered by a viral effector. NbHMP04-dependent host defenses against TYLCCNV are activated in the presence of copper ions, which enhances the interaction between NbHMP04 and NbNPR1, increasing NbNPR1 stability and up-regulating downstream gene expression. The TYLCCNB-encoded βC1 protein, however, interacts with NbHMP04 and induces NbHMP04 autophagic degradation to interrupt host immune responses. The TYLCCNV/TYLCCNB (βC1^Y110A^) mutant virus caused milder disease symptoms in the infected *Nicotiana benthamiana* plants. These findings provide novel insights into the mechanism by which copper enhances plant defense and reveal how geminiviruses have evolved to counter these defense responses during infection.

## Graphical abstract

**Figure:**
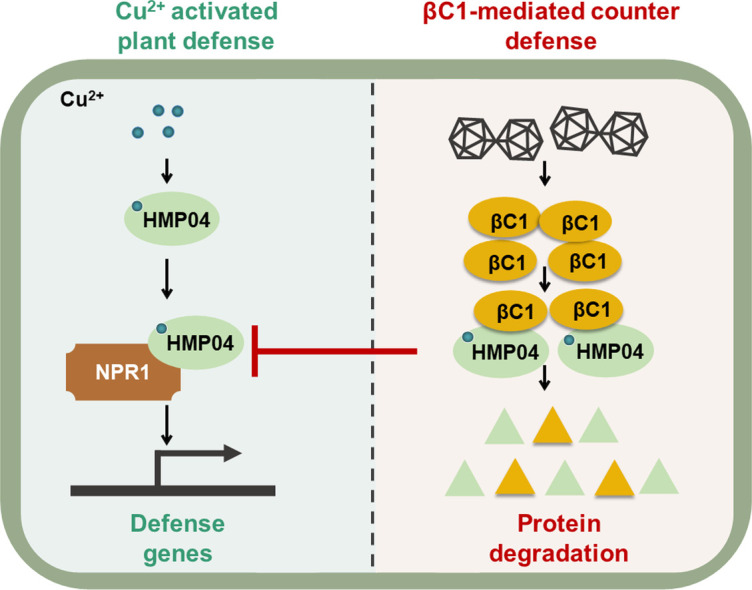

## 1 Introduction

Metal ions play important roles in plant growth and development, and their concentration and balance are essential for plant health [[1], [2], [3]]. The transport of metal ions into cells requires metallochaperone proteins, which are a family of metal ion transporters with the capacity to bind specific metal ions in eukaryotic cells [4]. The studies on metallochaperone proteins could be traced back to the 1990s with the discovery of metal-binding proteins in yeast, such as Lys7, Cox17, and Atx1, which were named copper chaperone proteins and found responsible for the transport of copper ions in *Saccharomyces cerevisiae* [[5], [6], [7]]. Plant metallochaperone-like proteins with high similarity to yeast metallochaperone proteins and specific metal-binding domains were identified in *Arabidopsis thaliana* (hereafter referred to as Arabidopsis), barley, and maize [[8], [9], [10]]. Metallochaperone-like proteins bind metal ions and transfer them to the target proteins via protein-protein interactions [11]. This protein family is widespread in plants and its members are characterized by having one or more HMA (Heavy Metal Associated) superfamily domains, which are necessary for their binding and transport of metal ions [12]. The HMA domain contains an MXCXXC motif, which is necessary for binding and transfer of ions [13,14], and two cysteine residues necessary for binding [15,16]. The HMA domain contains multiple cysteine and histidine residues, which form coordination bonds with metal ions to protect plants from metal toxicity [17,18]. The functions of metallochaperones include the regulation of plant growth, development, and immune processes. Expression of the rice metallochaperone OsHIPP24, for example, is induced by cadmium and copper ions, which has a positive regulatory effect on rice growth and development [19]. Arabidopsis AtHMA1 regulates the balance of zinc ion concentration in plant cells to maintain physiological processes, such as zinc ion absorption and biochemical metabolism [20]. Metallochaperone proteins have also been widely reported to be involved in plant resistance to abiotic and biotic stresses [21,22]. For example, the expression of TaHIPP1 in wheat was up-regulated by ABA exposure or wounding, and over-expression of TaHIPP1 in yeast significantly increased the rate of cell growth under copper and high salt stress [22]. In Arabidopsis, *AtHIPP3* acts as an upstream regulatory factor in the salicylic acid-dependent pathway of pathogen response [23], while *AtHIPP27* is a susceptibility factor required for the infection and development of beet cyst nematodes [24]. In *Nicotiana benthamiana*, NbHIPP26 interacts with TGB1, the movement protein of potato mop-top virus, to activate drought stress responses and promote long-distance movement of the virus [25]. Although the importance of metallochaperone proteins in plant defense has been widely recognized, their specific role in anti-viral defense, especially in host defense against geminiviruses, has not been well studied. Moreover, the molecular mechanisms underlying the metallochaperone proteins-mediated host immune responses are unknown.

NONEXPRESSION OF PR GENES 1 (NPR1) is a master regulator of the transcriptome induced by the defensive hormone salicylic acid (SA) [26]. When plants are under attack by pathogens, NPR1 is activated through a series of biochemical changes [27]; once activated, NPR1 leads to the upregulation of downstream genes, including *PR1*, followed by the synthesis and accumulation of PR1 proteins, ultimately enhancing plant disease resistance [28]. Several studies have shown that NPR1 is involved in plant immune responses against an array of different pathogens, including bacteria, fungi, and viruses. For example, NPR1 is activated in response to *Pseudomonas syringae* infection and promotes the expression of defense-related genes through SA signaling [29]. Heterologous expression of AtNPR1 also enables *Brassica juncea* to increase host resistance to fungal infection by *Sclerotinia sclerotiorum* and *Alternaria brassicae* [30]. In tobacco plants, the presence of a functional *NtNPR1* gene is necessary for the plant to exhibit resistance to TMV by regulating the expression of *PR* genes [31].

Geminiviruses cause devastating diseases in fields and economic losses in agriculture worldwide [32]. *Begomovirus* is the largest genus within the *Geminiviridae* family and the only one that comprises viruses with both monopartite and bipartite genomes [33,34]. Some monopartite begomoviruses associate with satellite molecules, which provide additional proteins acting as virulence factors [35]. Despite their small genome size and limited coding capacity, geminiviruses encode multifunctional proteins that enable their pathogenicity [36].

The βC1 protein encoded by betasatellites induces viral symptoms and interferes with host signaling through the direct interaction with host factors to create a suitable environment for the viral infection. Tomato yellow leaf curl China virus (TYLCCNV) is a monopartite geminivirus-associated betasatellite, Tomato yellow leaf curl China betasatellite (TYLCCNB). The βC1 encoded by TYLCCNB (TYLCCNB βC1) interacts with AS1 to suppress a subset of JA responses [37]. TYLCCNB βC1 has also been identified as a gene silencing suppressor: βC1 reverses transcriptional gene silencing (TGS) by interacting with and inhibiting S-adenosyl homocysteine hydrolase (SAHH), which reduces methylation of viral DNA [38]. Cotton leaf curl Multan virus (CLCuMuV) βC1 was found to interact with NbSKP1, a key component of SCF (SKP1, Cullin1, F-box) E3 ubiquitin ligase complexes to interfere with JA and GA signaling and enhance viral accumulation [39].

In this study, we describe that copper ions induce host defenses against TYLCCNV/TYLCCNB, and identified a metallochaperone-like protein NbHMP04 as essential for these responses. We further found that NbHMP04 interacts with NbNPR1 and enhances its stability via direct interaction. Interestingly, the interaction between NbHMP04 and NbNPR1 is enhanced in the presence of copper ions. TYLCCNB βC1, however, physically associates with NbHMP04 and antagonizes the interaction of NbHMP04 with NbNPR1, impairing the integrity of the NbHMP04/NbNPR1 complex and promoting the degradation of NbNPR1. Moreover, we pinpoint the 110th tyrosine in βC1 is important for its interaction with NbHMP04; expression of the NbHMP04-interaction compromised βC1 mutant does not influence NbNPR1 stability. Notably, we found that TYLCCNV/TYLCCNB escape the NbHMP04-mediated resistance by promoting the degradation of NbNPR1 by dissociating the NbHMP04/NbNPR1 complex via the action of βC1.

## 2 Materials and methods

### 2.1 Plant materials and growth conditions

Wild-type, *35S::βC1*, RFP-H2B, *35S::NbHMP04-Flag, nbhmp04* and *npr1 knock-out N. benthamiana* plants and *Solanum lycopersicum* cv. Ailsa Craig plants were cultivated in a greenhouse at 22 °C /18 °C (day/night), 16/8 h of light/dark, and 60% relative humidity. The plants were grown in a greenhouse for roughly four weeks in preparation for subsequent experiments.

### 2.2 Plasmids construction

All gene sequences were amplified using KOD One™ PCR Master Mix (cat: KMM-101, TOYOBO, Osaka, Japan) and then ligated with enzymatically cleaved linearized vectors using the homologous recombination method described in the ClonExpress II One Step Cloning Kit (cat: C112–02, Vazyme, Nanjing, China). The circular plasmid vector was processed with a restriction endonuclease. The *NbHMP04*^mHMA^ construct employed a point mutation strategy to alter the sequence GATTGTGATGGATGC to GATGGTGATGGAGGC, where two cysteines were converted into two glycines. Following this mutation, the modified sequence was assembled with an enzymatically-cleaved vector to generate the recombinant construct: 2 × 35S-NbHMP04^mHMA^-GFP. Plasmid TYLCCNB (βC1^Y110A^) was generated using a published protocol [40]. The *NbHMP04*-Cas9 construct was obtained as follows: using the CRISPR-P online website (http://crispr.hzau.edu.cn/CRISPR2/), two SgRNA sequences were designed and the dual-target knockout method was used. After annealing, the fragments were directly ligated into the BKG01 vector to obtain BKG01-*NbHMP04*. Supplementary Table 1 lists the primers utilized for plasmid generation in this study.

### 2.3 Agroinfiltration and viral inoculation

*A. tumefaciens* stains EHA105 harboring plasmids were grown in 5 mL of YEP liquid medium supplemented with 50 µg/mL kanamycin and 50 µg/mL Rifampicin liquid medium at 250 rpm and 28 °C overnight. Upon collecting the bacterial cells, they were resuspended using infiltration buffer (10 mM MgCl_2_, 10 mM MES pH = 7.5, 100 µM Acetosyringone). The OD_600_ of the resuspended bacteria was adjusted to OD_600_ = 1.0. After leaving the inoculum in the dark for 2–3 h, it was infiltrated on the back of *N. benthamiana* leaves. Samples were taken at 48 h post-infiltration.

### 2.4 Plant RNA extraction and RT-QPCR

Total plant RNA was extracted from *N. benthamiana* leaves using TRIzol reagent (Invitrogen, Carlsbad, CA, USA), according to the manufacturer's protocol. Subsequently, 500 ng of total RNA was reverse transcribed to cDNA using the ReverTra Ace qPCR RT kit (cat: FSQ-301, TOYOBO, Osaka, Japan). ChamQ SYBR Color qPCR Master Mix (cat: Q411–02, Vazyme, Nanjing, China) was employed for the qPCR reactions. RT-qPCR analysis was conducted using the LightCycler 480 (Roche, Rotkreuz, Switzerland) according to the manufacturer's protocol. Gene expression was relatively quantified through qPCR, and the relative expression levels were determined using the comparative CT method. *NbActin* RNA served as the internal control for normalization. The reactions were performed in triplicate, and the results were averaged.

### 2.5 Plant DNA extraction

Total plant DNA was extracted from *N. benthamiana* systemically infected leaves using the CTAB method [41]. According to the provided protocol, 300 ng of genomic DNA (gDNA) was utilized for qPCR assays to determine the accumulation of viral DNA. ChamQ SYBR Color qPCR Master Mix (cat: Q411–02, Vazyme, Nanjing, China) was employed for the qPCR reactions.

### 2.6 Bimolecular complementary fluorescence

*A.tumefaciens* mixtures carrying the appropriate BiFC constructs were subsequently co-infiltrated into *N. benthamiana* leaves. The infiltrated portion was imaged at 48 h post-infiltration. The YFP excitation light was at 514 nm and the emission was detected at 520–560 nm. The RFP excitation light was at 561 nm and the emission was detected at 560–620 nm. The images were taken by a laser scanning confocal microscope FV3000 (Olympus, Tokyo, Japan) and analyzed with FV31S-DT (Olympus).

### 2.7 Protein extraction and western blot

Approximately 0.1 g *N. benthamiana* leaf samples were extracted with buffer containing pH 7.5 Tris–HCl, 150 mM; Urea, 6 M; SDS, 2%; β-Mercaptoethanol, 2%. Immunoblotting was carried out as described previously. The blotted signal was visualized using chemiluminescence (GE Healthcare, Chicago, IL, USA) according to the manufacturer's manual. The primary antibodies against GFP (cat: AE012, ABclonal, Wuhan, China), FLAG tag (cat: F1804, Sigma, St. Louis, MO, USA), Actin (cat: AC009, ABclonal, Wuhan, China) and the specific antibodies against NbHMP04 were generated by HUABIO (Hangzhou, China). Primary antibodies against TYLCCNV CP were generated in our lab.

### 2.8 Hydroponic culture

Seeds of *N. benthamiana* and *S. lycopersicum* were germinated and grown to the three-leaf stage on seedling blocks, transplanted into a hydroponic tank, and cultured for one week using 1/2 Hoagland's culture solution. Then, the original culture solution was washed away and replaced with a modified full-strength Hoagland nutrient solution with varied copper ions concentrations (0.3 µM as low concentration and 3 µM as high concentration) for three to four weeks each. The formula of the modified full-strength Hoagland nutrient solution was as follows (mM): Ca(NO_3_)_2_·4H_2_O, 4.0; KNO_3_, 6.0; NH_4_H_2_PO_4_, 1.0; MgSO_4_·7H_2_O, 2.0; MnSO_4_·4H_2_O, 1.0 × 10^−^^2^; H_3_BO_3_, 5.0 × 10^−2^; FeSO_4_·7H_2_O, 5.1 × 10^−2^; C_10_H_14_N_2_O_8_Na_2_·2H_2_O, 5.1 × 10^−2^; ZnSO_4_·7H_2_O, 8.0 × 10^−^^4^; CuSO_4_·5H_2_O, 5.0 × 10^−^^4^ and (NH_4_)_6_Mo_7_O_24_·4H_2_O, 2.0 × 10^−^^5^.

### 2.9 Yeast two-hybrid

Combination of plasmids AD-NbHMP04 and BD-NbNPR1, AD-NbHMP04 and BD-βC1, AD-NbHMP04 and BD-βC1 mutants, were co-transformed into *Saccharomyces cerevisiae* strain Gold. AD-T and BD-p53 were transformed into *S. cerevisiae* strain Gold to serve as a positive control, AD-T and BD-Lam, AD and BD-NbNPR1/BD-βC1/BD-βC1 mutants or AD-NbHMP04 and BD were co-transformed into *S. cerevisiae* strain Gold to serve as a negative control. All transformants were grown at 30 °C for 72 h on the SD medium lacking Leu and Trp and then transferred to the medium lacking Leu, Trp, His, and Ade.

### 2.10 Co-immunoprecipitation

About 0.5 g of the infiltrated area of *N. benthamiana* leaves were collected and the proteins were extracted using Co-IP buffer (pH 7.5 Tris–HCl, 20 mM; NaCl, 150 mM; MgCl_2_, 5 mM; DTT, 5 mM; pH 8.0 EDTA, 2 mM; glycerol, 2%; Triton X-100, 0.1%, cocktail (MCE, Monmouth Junction, NJ, USA), 1 pellet/50 mL) as previously described [42]. Anti-Flag Magnetic Beads (cat: M8823–1ML, Sigma, St. Louis, MO, USA) or Anti-HA Magnetic Beads (cat: 88,836, Thermo Scientific, Waltham, MA, USA) for 2 h in a 4 °C refrigerator. Thermo DynaMag™−2 (cat: 12321D, Thermo Scientific, Waltham, MA, USA) was used to adsorb the beads. For the Cu binding assay, special PureCube Cu-NTA MagBeads (cat: 31,501-Cu, Cube Biotech, Rheinland, Germany) were used, and the Co-IP buffer was without EDTA.

### 2.11 Semi-*in vivo* degradation

ATP (cat: A7699, Sigma, Saint Louis, USA) was dissolved in ddH_2_O and utilized at a concentration of 20 mM (Sigma, Saint Louis, USA). 100 µM MG132 (cat: C2211, Sigma, Saint Louis, USA), 50 µM E64d (cat: HY-100,229, Sigma, Saint Louis, USA), and 100 µM cycloheximide (CHX) (cat: HY-12,320 MCE, Monmouth Junction, NJ, USA) were dissolved in 0.1% DMSO. Every sample was taken at 24 h post-infiltration for semi-*in vivo* protein degradation analysis. The samples were extracted separately with NBI buffer (pH 8.0 Tris-MES, 50 mM; Sucrose, 0.5 M; MgCl_2_, 1 mM; pH 8.0 EDTA, 10 mM; and DTT, 10 mM) with the stated doses of ATP, CHX, and MG132, E64d, or DMSO (control). The mixtures were incubated at room temperature with agitation in an Eppendorf Thermomixer (Eppendorf, Hamburg, Germany) for 0 h, 2 h, and 4 h. Prior to immunoblot analysis, samples were withdrawn and reactions were stopped by adding SDS sample buffer and boiling for 5–10 min.

### 2.12 Analysis of AtHMPS genes and phylogenetic tree construction

AtHMPs genes were identified by searching the *Arabidopsis thaliana* genomic database with the sequence of *NbHMP04* and referring to the genes as Tehseen et al. [43] previously described. Phylogenetic analyses were performed using the maximum likelihood method by MEGA X.

## 3 Results

### 3.1 NbHMP04 plays a critical role in plant defense against TYLCCNV/TYLCCNB

**Fig. 1:**
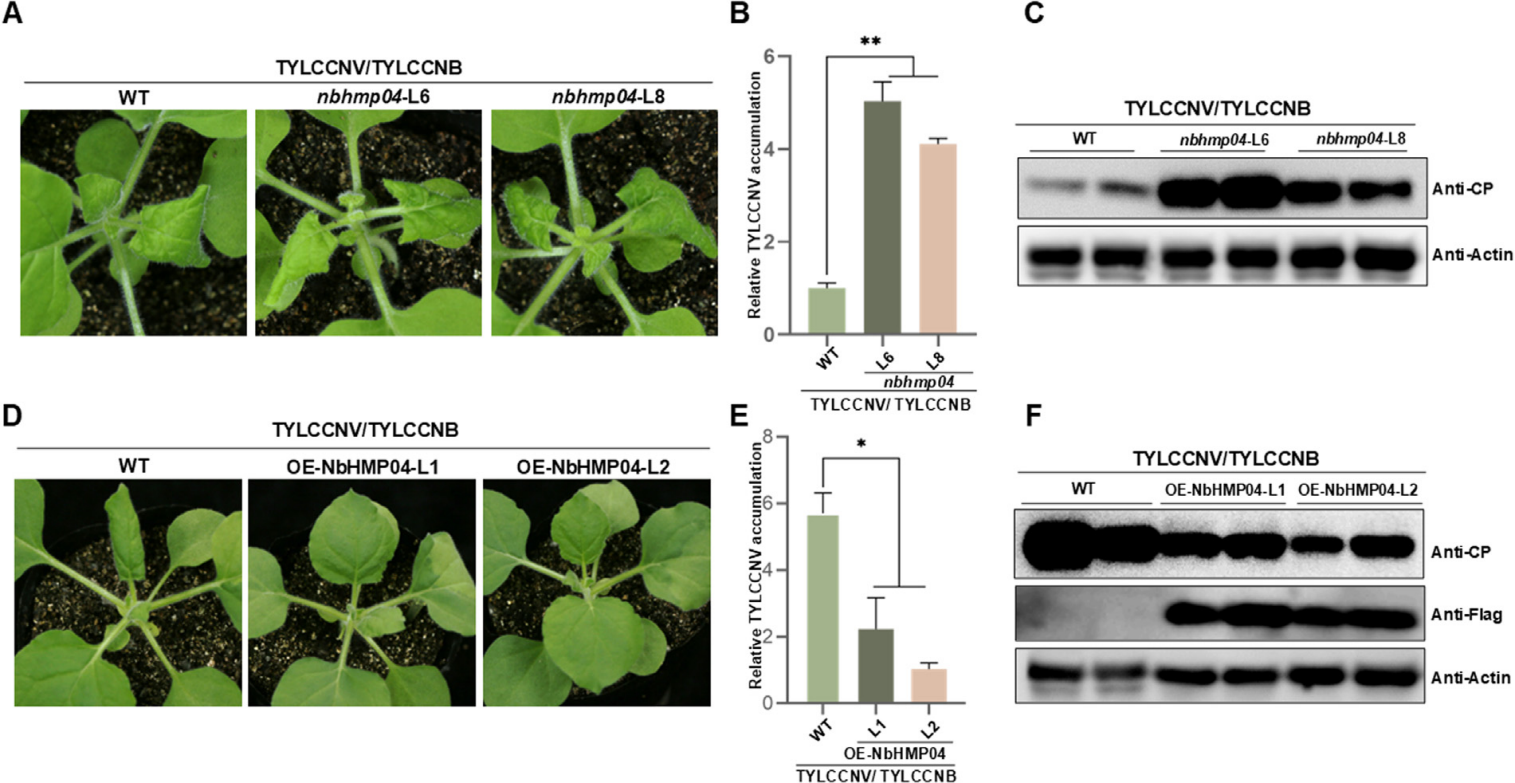
**NbHMP04 promotes resistance of*****N. benthamiana*****plants to the infection by TYLCCNV/TYLCCNB infectious clones.** (A) Symptoms of wild-type, *NbHMP04*-L6, and *NbHMP04*-L8 *N. benthamiana* plants infected with TYLCCNV/TYLCCNB at 6 days post-inoculation (dpi). (B) Quantitative real-time PCR (qPCR) showing accumulation of TYLCCNV *CP* in systemically infected leaves from (A). Data are presented as means ± SD of three biological replicates. Statistical analyses were performed using the Student's *t*-test. **, *p* < 0.01. *NbActin* was used as the internal reference gene. (C) Western blot showing protein accumulation of TYLCCNV CP in systemically infected leaves of wild-type, *nbhmp04*-L6, and *nbhmp04*-L8 *N. benthamiana* from (A). NbActin acts as the loading control. (D) Symptoms of wild-type, OE-NbHMP04-L1, and OE-NbHMP04-L2 *N. benthamiana* plants infected with TYLCCNV/TYLCCNB at 6 dpi. (E) qPCR showing accumulation of TYLCCNV *CP* in systemically infected leaves from (D). Data are presented as means ± SD of three biological replicates. Statistical analyses were performed using the Student's *t*-test. *, *p* < 0.05. *NbActin* was used as the internal reference gene. (F) Western blot showing protein accumulation of TYLCCNV CP in systemically infected leaves of wild-type, *NbHMP04*-L6, and *NbHMP04*-L8 *N. benthamiana* from (D). NbActin acts as the loading control.

To investigate the relationship between HMPs and the pathogenicity of geminiviruses, we performed the transcriptome sequencing of wild-type (WT) or *35S::βC1 Nicotiana benthamiana* plants (a transgenic *N. benthamiana* overexpressing the symptom-determinant factor of TYLCCNV). Results showed that several heavy metal-related genes were up-regulated in *35S::βC1 N. benthamiana* plants compared with wild-type and seven heavy metal-related genes with the most significant up-regulated expression were selected in *35S::βC1 N. benthamiana* plants for analysis (Fig. S1A). Particularly, *NbHIPP26,* as a homolog of Arabidopsis *AtHIPP26,* has been reported to be involved in virus infection [25], so *NbHMP04*, which is closely related to *AtHIPP26*, was selected for further study. As expected, quantitative reverse transcription PCR (qPCR) results showed that TYLCCNV/TYLCCNB infection indeed induced the upregulation of *NbHMP04* in *N. benthamiana* plants at 10 days post-inoculation (dpi) (Fig. S1B). To study whether this induction of *NbHMP04* has biological relevance, we performed CRISPR-Cas9-mediated genome editing for the knockout of *NbHMP04,* and two independent lines were obtained. Sequence analysis revealed that both knock-out lines, named *nbhmp04*-L6 and *nbhmp04*-L8, had 8 nucleotides missing in the *NbHMP04* coding region (Fig. S1C-D). Then we inoculated wild-type (WT), *nbhmp04-*L6, and *nbhmp04-*L8 *N. benthamiana* plants with TYLCCNV/TYLCCNB, strikingly, *nbhmp04* knock-out plants were hypersensitive to TYLCCNV/TYLCCNB infection, displaying more severe symptoms and higher viral accumulation (Fig. 1A–C). These results suggest that *NbHMP04* plays a critical role in host resistance against TYLCCNV/TYLCCNB. To further confirm the biological function of *NbHMP04* in anti-geminiviral resistance, *NbHMP04* cDNA was introduced into a binary plant expression vector under the control of cauliflower mosaic virus (*CaMV*) *35S* constitutive promoter to generate *35S::NbHMP04-Flag* transgenic *N. benthamiana* plants, overexpressing this gene, referred as OE-NbHMP04-L1, OE-NbHMP04-L2 (Fig. S1E-F). As results showed, transgenic *N. benthamiana* plants overexpressing *NbHMP04* exhibited enhanced resistance to TYLCCNV/TYLCCNB infection, displaying milder symptoms in systemic leaves and lower viral accumulation (Fig. 1D–F). Taken together, these results indicate that NbHMP04 plays an important role in the restriction of TYLCCNV/TYLCCNB infection.

### 3.2 NbHMP04 is a master regulator of copper ions-mediated plant antiviral defense

**Fig. 2:**
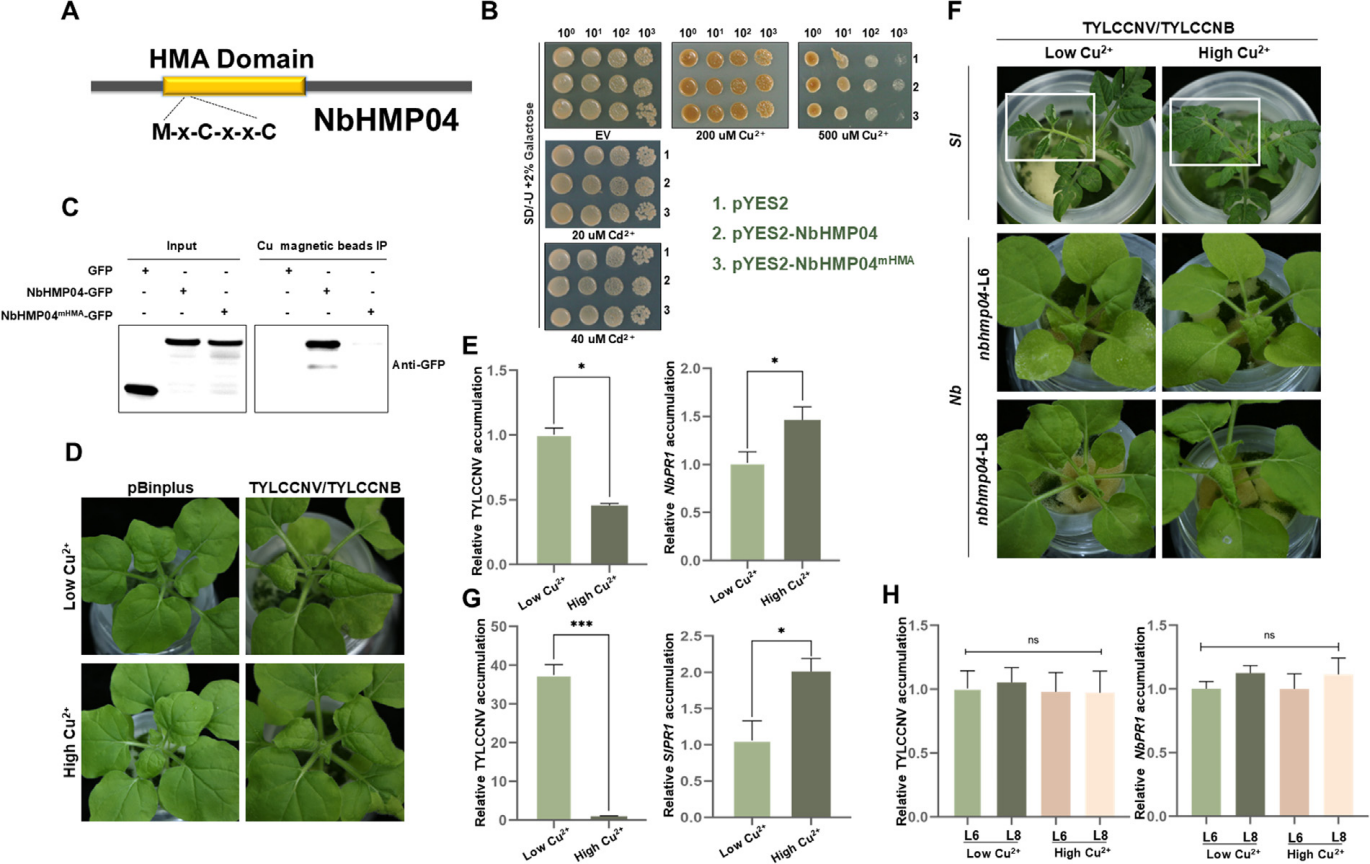
**NbHMP04 binds to copper ions to stimulate plant immunity.** (A) Schematic representation of the conserved domains of NbHMP04 protein. HMA represents heavy metal associated domain; M represents Met; C represents Cys; x represents any amino acid. (B) Copper (Cu) and cadmium (Cd) tolerance of NbHMP04- or NbHMP04^mHMA^-transformed yeast cells. Yeast cells transformed with vector pYES2 (as a negative control), pYES2-NbHMP04, or pYES2- NbHMP04^mHMA^ were grown in SD/-Urea with 2% galactose for 72 h (h), and their OD_600_ was adjusted to 1, 0.1, 0.01, 0.001; the diluted cultures were spotted onto medium supplemented with 0 µM,20 µM, 40 µM Cd^2+^, or with 0 µM, 200 µM, 500 µM Cu^2+^. (C) Cu-NTA beads pull down the NbHMP04 protein. Total protein was extracted from GFP-, NbHMP04-GFP-, or NbHMP04^mHMA^-GFP-expressing leaves, then incubated with Cu-NTA Beads for 2 h. Anti-GFP antibodies were used to detect the pulled-down proteins. GFP was used as the negative control. (D) Symptoms of wild-type *N. benthamiana* plants grown in low (0.3 µM) or high (3 µM) Cu^2+^ hydroponic solution inoculated with TYLCCNV/TYLCCNB infectious clones or pBinplus (as a negative control) at 6 days post-inoculation (dpi). (E) Quantitative real-time PCR (qPCR) showing accumulation of TYLCCNV *CP* (left part) and *NbPR1* (right part) in systemically infected leaves from (D). Data are presented as means ± SD of three biological replicates. Statistical analyses were performed using the Student's *t*-test. *, *p* < 0.05. *NbActin* was used as the internal reference gene. (F) Symptoms of *Solanum lycopersicum* plants (upper panel) or *nbhmp04*-L6 and *nbhmp04*-L8 *N. benthamiana* plants (lower panel) infected with TYLCCNV/TYLCCNB at 6 dpi. Plants were grown in low or high Cu^2+^ hydroponic solution. (G) qPCR showing accumulation of TYLCCNV *CP* (left panel) and *SlPR1* (right panel) in systemically infected leaves from (upper panel of F). Data are presented as means ± SD of three biological replicates. Statistical analyses were performed using the Student's *t*-test. *, *p* < 0.05, ***, *p* < 0.001. *SlActin* was used as the internal reference gene. (H) qPCR showing accumulation of TYLCCNV *CP* (left panel) and *NbPR1* (right panel) in systemically infected leaves from (lower panel of F). Data are presented as means ± SD of three biological replicates. ns, not significant. *NbActin* was used as the internal reference gene.

NbHMP04 contains an HMA domain, and the predicted structure of the protein has a high similarity with that of AtHMPs (Fig. 2A and S2A-B). Previous studies reported that HMPs protect plants from metal toxicity by binding heavy metal ions directly [17,18]. To elucidate whether NbHMP04 could bind metal ions specifically, we tested the sensitivity of yeast producing NbHMP04 protein to cadmium and copper ions. Interestingly, yeast expressing NbHMP04 were insensitive to copper ions, whereas plants expressing a predicted NbHMP04 mutant with compromised metal ion binding ability (due to mutation of the cysteine to glycine in the HMA MxCxxC motif) exhibited hypersensitivity (Fig. 2B). These results suggest that NbHMP04 might indeed bind copper ions, therefore enhancing the tolerance of yeast to copper ions stress. To determine whether NbHMP04 has the capacity to bind copper ions directly, Cu-NTA MagBeads were used for copper ions-binding assays. NbHMP04-GFP, NbHMP04^mHMA^-GFP, and GFP (as a negative control) were expressed in leaves of *N. benthamiana* plants individually, extracted and purified via copper affinity, and the copper-binding ability of NbHMP04 was assessed. Immunoblot results showed that NbHMP04 could be enriched by Cu-NTA MagBeads, but mutant NbHMP04^mHMA^ and the negative control were not (Fig. 2C). These results support the idea that NbHMP04 is a metallochaperone-like protein and binds copper ions directly. A recent study showed that copper ions can trigger broad-spectrum resistance to viruses in rice [44]. The copper-binding capacity of NbHMP04 raises the possibility that NbHMP04 contributes to copper ions-mediated host defense. To test this hypothesis, we confirmed that copper ions negatively affect TYLCCNV/TYLCCNB infection. Indeed, *N. benthamiana* plants grown in culture solutions containing higher concentrations of copper ions showed milder symptoms and lower viral accumulation when inoculated with TYLCCNV/TYLCCNB (Fig. 2D, E left panel and Fig. S3A upper panel). Consistent with the enhanced resistance against TYLCCNV/TYLCCNB, the *PR1* gene was up-regulated in *N. benthamiana* plants grown in culture solutions containing higher concentrations of copper ions (Fig. 2E right panel). TYLCCNV/TYLCCNB-infected *Solanum lycopersicum* plants grown in culture solutions containing higher concentrations of copper ions displayed a similar phenotype, showing milder symptoms, lower viral accumulation, and higher *PR1* transcript accumulation (Fig. 2F upper panel, Fig. S3A middle panel and Fig. 2G). These results suggest that copper ions trigger plant defenses against TYLCCNV/TYLCCNB. To investigate the role of NbHMP04 in plant immunity to TYLCCNV/TYLCCNB infection, we inoculated *nbhmp04* knock-out *N. benthamiana* plants grown in culture solutions containing low or high concentrations of copper ions with TYLCCNV/TYLCCNB. Results showed that no significant difference could be detected in symptoms or viral accumulation between the *nbhmp04* knock-out *N. benthamiana* plants grown at low or high Cu^2+^concentrations, which correlated with a lack of differential *PR1* expression (Fig. 2F lower panel, Fig. 2H and Fig. S3A lower panel). These results indicate that NbHMP04 is essential for copper ions-mediated host immunity to TYLCCNV/TYLCCNB. Consistent with these results, both wild-type and *nbhmp04* knock-out *N. benthamiana* plants exhibited similarly severe viral symptoms at low copper concentrations (Fig. S3B), while under hydroponic conditions with high concentrations of copper ions, *nbhmp04* knock-out *N. benthamiana* still exhibited severe viral symptoms but wild-type *N. benthamiana* did not (Fig. S3B). Protein level measurements reinforced the previous conclusion: under low Cu^2+^ hydroponic conditions, the *nbhmp04* knock-out *N. benthamiana* accumulated higher viral titers than wild-type *N. benthamiana*. Under high Cu^2+^ hydroponic conditions, lower viral titers were observed in wild-type *N. benthamiana* compared to low Cu^2+^ hydroponic conditions; viral titer did however not change in a copper-dependent manner in *nbhmp04* knock-out *N. benthamiana* (Fig. S3C). Quantification of viral DNA accumulation and *NbPR1* expression level led to similar results (Fig. S3D). In conclusion, NbHMP04 is a host factor that mediates plant copper ions-mediated resistance to TYLCCNV/TYLCCNB infection.

### 3.3 NbHMP04 protects NbNPR1 from degradation through direct interaction

**Fig. 3:**
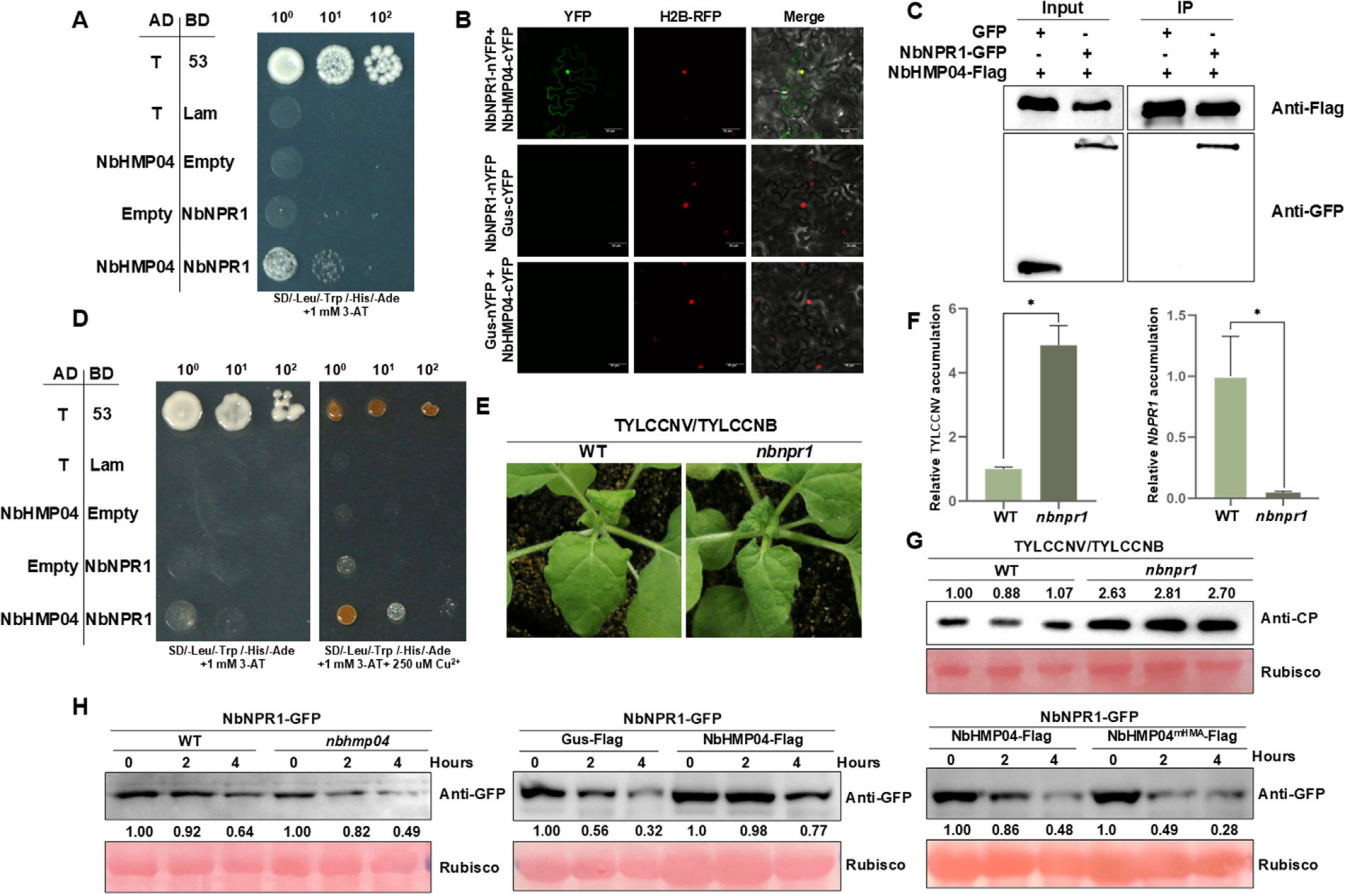
**NbHMP04 interacts with NbNPR1 to enhance plant defense.** (A) NbHMP04 and NbNPR1 interact in yeast. AD represents activation domain; BD represents binding domain. The interaction between the SV40 large T antigen (T) and the tumor suppressor p53 acts as a positive control; empty AD- and BD-containing vectors (AD and BD, respectively) acts as a negative control. Yeast cells are grown on SD/-Leu/-Trp/-His/-Ade plates; 3-AT: 3-Amino-1,2,4-triazole. (B) BiFC assay of the interaction of NbHMP04-cYFP and NbNPR1-nYFP, NbHMP04-cYFP and Gus-nYFP, or Gus-cYFP and NbNPR1-nYFP in H2B-RFP transgenic *N. benthamiana* leaves. Confocal images were captured at 48 h post-infiltration (hpi), and H2B-RFP was used as a nuclear marker. YFP excitation light at 514 nm and RFP excitation light at 561 nm, Scale bar: 50 µm. (C) Co-IP analysis of the interaction between NbHMP04 and NbNPR1. Total protein extracts were immunoprecipitated with anti-Flag beads followed by immunoblotting using anti-Flag or anti-GFP antibodies. GFP acts as the negative control. (D) Interaction between NbHMP04 and NbNPR1 in yeast grown in 0 µM or 250 µM Cu^2+^-containing SD/-Leu/-Trp/-His/-Ade plates. (E) Symptoms of wild-type and *npr1 N. benthamiana* plants infected with TYLCCNV/TYLCCNB at 6 days post-inoculation (dpi). (F) Quantitative real time PCR showing accumulation of TYLCCNV *CP* (left panel) and *NbPR1* (right panel) in systemically infected leaves from (E). Data are presented as means ± SD of three biological replicates. Statistical analyses were performed using the Student's *t*-test. *, *p* < 0.05. *NbActin* was used as the internal reference gene. (G) Western blot showing protein accumulation of TYLCCNV CP in systemically infected leaves of wild-type and *npr1 N. benthamiana* from (E). The corresponding Ponceau S staining of the large Rubisco subunit acts as the loading control. The quantitative analysis of western blot results was performed by ImageJ. Numbers in figure represent the results of quantitative analysis. (H) Semi-*in vivo* protein stability assay of NbNPR1-GFP in wild-type and *nbhmp04 N. benthamiana* leaves (left panel), *N. benthamiana* leaves co-infiltrated with NbHMP04-Flag or Gus-Flag (as a negative control) (middle panel), or *N. benthamiana* leaves co-infiltrated with NbHMP04-Flag or NbHMP04^mHMA^-Flag (right panel)*.* NbNPR1-GFP was detected by immunoblotting with anti-GFP antibody at different time points after 100 µM CHX treatments in the presence of 20 mM ATP. The hours represent the time (hours) after adding ATP. 100 µM CHX is used to inhibit the synthesis of new protein. 20 mM ATP is used to provide the primary energy. The corresponding Ponceau S staining of the large Rubisco subunit acts as the loading control. The quantitative analysis of western blot results was performed by ImageJ. Numbers in figure represent the results of quantitative analysis.

To elucidate the molecular mechanism by which NbHMP04 exerts its function in the promotion of in-host defenses, we screened a *N. benthamiana* cDNA library by yeast two-hybrid (Y2H) to identify NbHMP04-interacting proteins. Among the NbHMP04-interacting clones, one cDNA encoding NONEXPRESSION OF PR GENES 1 (NPR1) was recovered. We next validated the interaction between NbHMP04 and NbNPR1 by Y2H, bimolecular fluorescence complementation, and co-immunoprecipitation (Co-IP) assays (Fig. 3A–C). It has been previously reported that *NPR1* binds to salicylic acid to activate the immune pathway in Arabidopsis in a copper ions-dependent manner [45]. To investigate the influence of copper on the interaction between NbHMP04 and NbNPR1, we added copper ions to the Y2H system; interestingly, an enhancement in the interaction between NbHMP04 and NbNPR1 could be observed as the concentration of copper ions increased (Fig. 3D). To determine the biological relevance of NbNPR1 for the TYLCCNV/TYLCCNB infection, we incubated *nbnpr1* knock-out plants with TYLCCNV/TYLCCNB. Notably, loss of NbNPR1 led to exacerbated symptoms, increased viral accumulation, and lower *NbPR1* transcripts (Fig. 3E–G), indicating that this protein exerts a potential role in antiviral defense. To elucidate the biological significance of the interaction between NbHMP04 and NbNPR1, we performed a semi-*in vivo* degradation assay, which demonstrated that NbHMP04 increases NbNPR1 protein stability in a copper ions-binding manner (Fig. 3H). Taken together, our results show that copper ions enhance the interaction between NbHMP04 and NbNPR1, which increases the stability of NbNPR1, ultimately promoting NbNPR1-mediated defense responses against TYLCCNV/TYLCCNB.

### 3.4 βC1 encoded by TYLCCNB promotes the degradation of NbHMP04 to counter host defenses

**Fig. 4:**
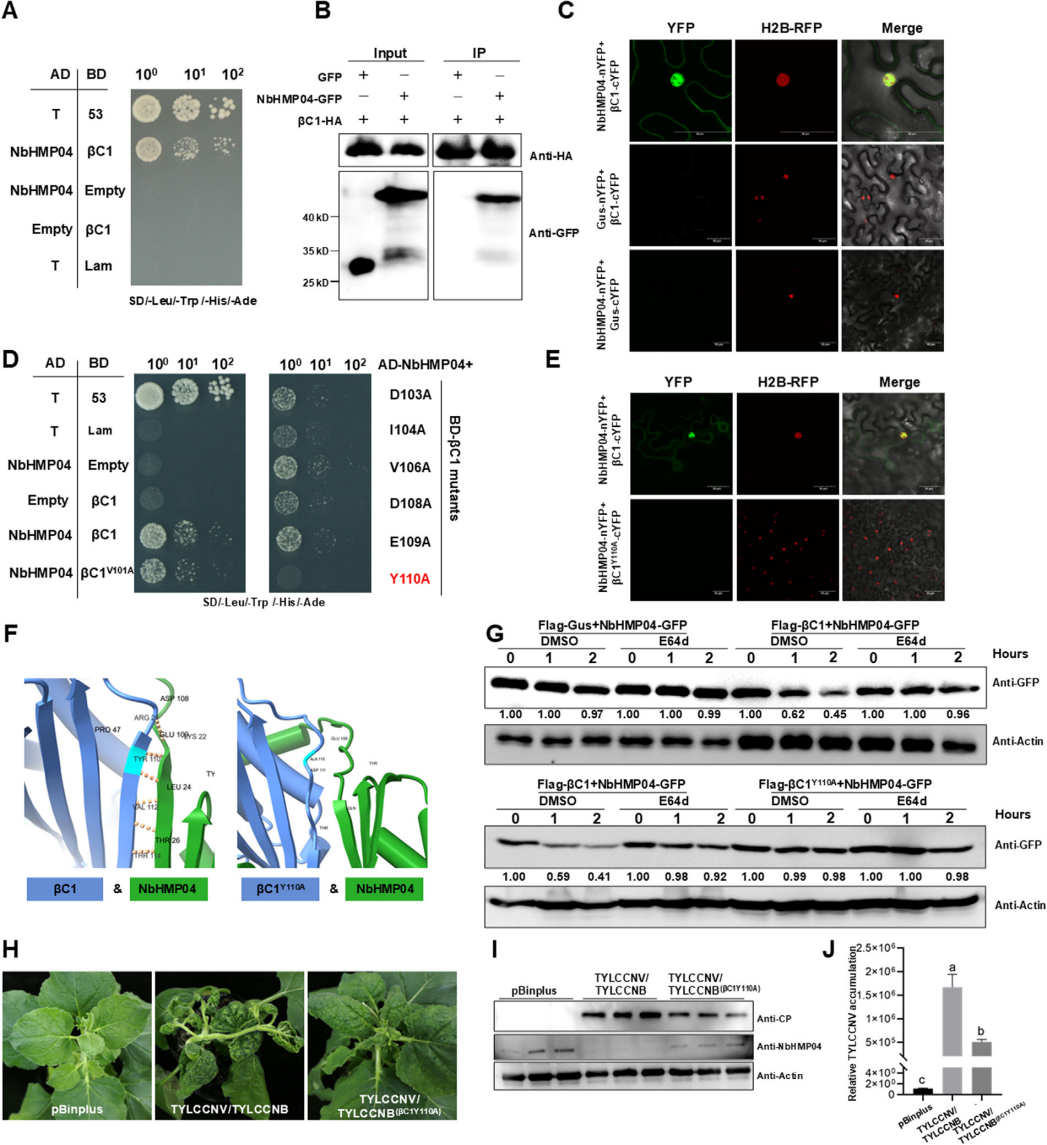
**βC1 interacts with NbHMP04 and induces its degradation.** (A) NbHMP04 and βC1 interact in yeast. AD represents activation domain; BD represents binding domain. The interaction between the SV40 large T antigen (T) and the tumor suppressor p53 acts as positive control; empty AD- and BD-containing vectors (AD and BD, respectively) are used as negative control. Yeast cells are grown on SD/-Leu/-Trp/-His/-Ade plates. (B) Co-IP analysis of the interaction between NbHMP04 and βC1. Total protein extracts were immunoprecipitated with anti-HA beads followed by immunoblotting using anti-HA or anti-GFP antibodies. GFP acts as the negative control. (C) BiFC assay of the interaction between NbHMP04-nYFP and βC1-cYFP, NbHMP04-nYFP and Gus-cYFP, or Gus-nYFP and βC1-cYFP in H2B-RFP transgenic *N. benthamiana* leaves. Confocal images were captured at 48 h post-infiltration (hpi); H2B-RFP was used as nuclear marker. Scale bar: 50 µm. (D) Interaction between point mutant versions of βC1 and NbHMP04. The capital letters in the figure represents the amino acids. V: Val, A: Ala, D: Asp; I: Ile, E: Glu; Y: Tyr. (E) BiFC assay of the interaction between NbHMP04-nYFP and βC1-cYFP, or NbHMP04-nYFP and βC1-cYFP^Y110A^ in H2B-RFP transgenic *N. benthamiana* leaves. Confocal images were captured at 48 hpi. H2B-RFP was used as nuclear marker. Scale bar: 50 µm. (F) Protein structure prediction of NbHMP04 and βC1 interaction complex or NbHMP04 and βC1 non-interaction complex using AlphaFold II, and visualized using RCSB Pub view. Yellow dashed lines indicate hydrogen bonds or non-covalent bonds. (G) Semi-*in vivo* protein stability assay of NbHMP04-GFP co-infiltrated with Flag-βC1 or Flag-Gus (as a negative control) into *N. benthamiana* leaves (the upper panel) or NbHMP04-GFP co-infiltrated with Flag-βC1 or Flag-βC1^Y110A^ into *N. benthamiana* leaves (the lower panel). NbHMP04-GFP protein level was detected by immunoblotting with anti-GFP antibody at different time points after 100 µM CHX treatments in the presence of 20 mM ATP. The Hours represents the time (hours) after adding 20 mM ATP. 100 µM CHX is used to inhibit the synthesis of new protein. 20 mM ATP is used to provide the primary energy, 50 µM E64d is used to inhibit degradation by autophagy and 0.1% DMSO as control. NbActin acts as the loading control. The quantitative analysis of western blot results was performed by ImageJ. Numbers in figure represent the results of quantitative analysis. (H) Symptoms of wild-type *N. benthamiana* plants inoculated with TYLCCNV/TYLCCNB infectious clones, TYLCCNV/TYLCCNB^(βC1Y110A)^ mutant infectious clones, or pBinplus (as a negative control) at 24 days post-inoculation (dpi). (I) Western blot showing protein accumulation of TYLCCNV CP and endogenous NbHMP04 in systemically infected leaves from (H). NbActin acts as the loading control. (J) Quantitative real-time PCR showing accumulation of *TYLCCNV CP* from (H) systemically infected leaves. Data are presented as means ± SD of three biological replicates. The letters a, b and c showing statistically significant differences between variables. *NbActin* was used as the internal reference gene.

Although TYLCCNV/TYLCCNB infection upregulates the *NbHMP04* transcript (Fig. S1A), western blot results show that endogenous NbHMP04 protein accumulation is lower in infected leaves (Fig. S4A), suggesting that a virus-encoded protein might be targeting it to hamper defense. To investigate this idea, we assessed the interaction between NbHMP04 and viral effectors by Y2H. Intriguingly, we found that TYLCCNB βC1 interacts with NbHMP04 (Fig. 4A) in yeast, but also in Co-IP and BiFC assays (Fig. 4B–C). To determine the effect of βC1 on the stability of NbHMP04, we detected the NbHMP04 accumulation in wild-type (WT) and *35S::βC1* transgenic *N. benthamiana* plants. Immunoblot results showed that the NbHMP04 accumulation is significantly lower in *35S::βC1* transgenic *N. benthamiana* plants than in WT *N. benthamiana* plants (Fig. S4B). The decreased NbHMP04 accumulation in *35S::βC1* transgenic *N. benthamiana* plants raises the possibility that βC1 might promote the degradation of this protein. To test this hypothesis, a degradation assay *in vivo* was performed. We added the autophagy pathway inhibitor E64d and the 26S proteasome inhibitor MG132 to infiltrated leaves. We found that when the autophagy inhibitor was added, it could significantly inhibit the degradation of NbHMP04 (Fig. S4C upper panel). However, after the addition of MG132, the inhibitory effect was not significant (Fig. S4C lower panel). These results showed that βC1 may promote NbHMP04 autophagic degradation. To further elucidate whether βC1 influences NbHMP04 stability through direct interaction, we generated ten βC1 partial versions and repeated the Y2H assays, and found a 10-amino acid stretch in βC1 (101–110 aa) that was crucial for the interaction (Fig. S4D). We then carried out site-directed mutagenesis to pinpoint the key residue(s) involved in the association. Y2H and BiFC assays showed that 110th tyrosine in βC1 was vital for the interaction with NbHMP04 (Fig. 4D–E). Consistent with the experimental results, the 110th tyrosine of βC1 was predicted as the key point for the interaction by using AlphaFold II (Fig. 4F). To study whether the interaction between βC1 and NbHMP04 has biological relevance, we detected the NbHMP04 protein stability in the presence of βC1 or a βC1^Y110A^ mutant via a semi-*in vivo* degradation assay. Immunoblot results showed the presence of βC1 significantly accelerated the degradation of NbHMP04. After adding the E64d inhibitor, the degradation phenomenon of NbHMP04 disappeared. When replaced by a non-interacting βC1^Y110A^, the promotion of NbHMP04 degradation by βC1 disappeared (Fig. 4G). We also constructed a TYLCCNV/TYLCCNB (βC1^Y110A^) infectious clone mutant harboring βC1^Y110A^, which loses the ability to interact with NbHMP04. Notably, the symptoms caused by TYLCCNV/TYLCCNB (βC1^Y110A^) were milder than those caused by TYLCCNV/TYLCCNB (Fig. 4H), which correlated with both lower endogenous NbHMP04 accumulation and viral accumulation at 24 days post-inoculation (dpi) (Fig. 4I–J). Those results suggest that βC1 counters NbHMP04-mediated defenses by inducing NbHMP04 autophagic degradation via direct interaction.

## 4 Discussion and conclusion

**Fig. 5:**
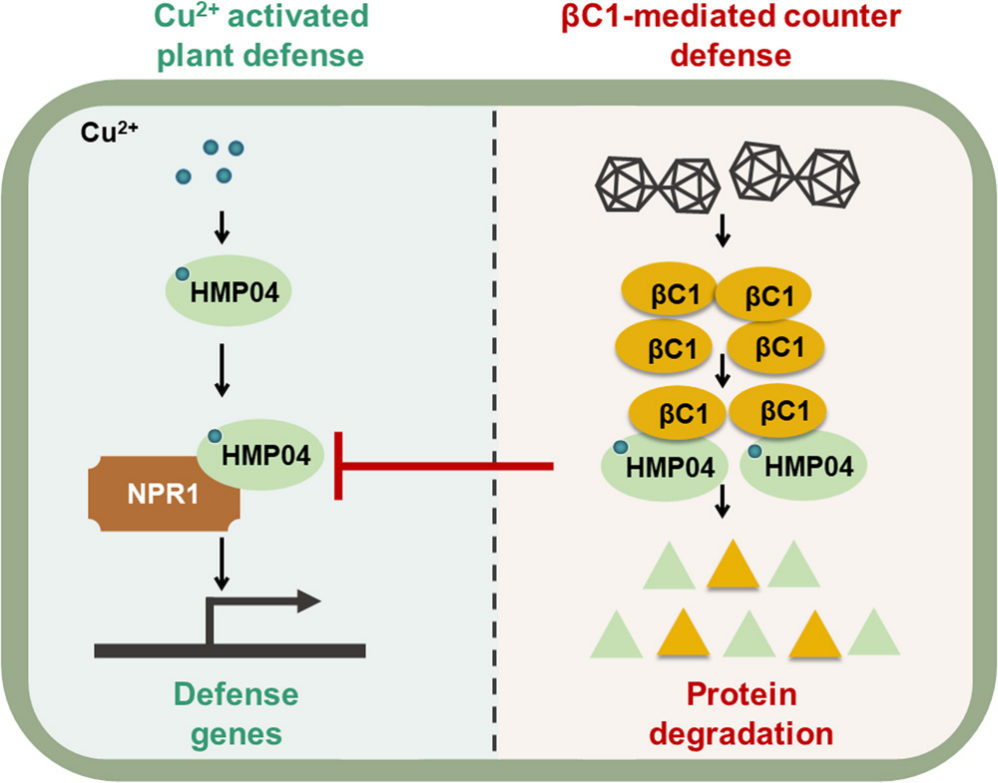
Proposed working model for the role of NbHMP04 in anti-viral resistance. Upon viral infection, *NbHMP04* is up-regulated, and NbHMP04 interacts with NbNPR1 to enhance its stability in a copper-dependent manner, thereby enhancing plant defense. Geminiviruses, however, might deploy counter-defense strategies, exemplified here in the geminivirus-encoded βC1 protein, which interacts with NbHMP04 and promotes its degradation through autophagy, therefore hampering plant defense.

Geminiviruses have been subjected to numerous studies in the past decades, and a plethora of aspects of the plant-virus interaction has been unveiled; we are, however, still far from a full understanding of the infection. In this study, we discovered a metallochaperone-like protein, NbHMP04, which can bind copper, through the analysis of geminivirus-induced transcriptional changes. By employing genetics, we discovered that NbHMP04 is a host factor that contributes to resistance against geminiviral infection. Further analysis showed that high levels of copper ions increase the host plant's resistance to TYLCCNV/TYLCCNB in a NbHMP04-dependent manner. Next, we found that NbHMP04 interacts with the immune regulator NbNPR1. Semi-*in vivo* degradation assays demonstrated that the interaction between NbHMP04 and NbNPR1 can enhance the stability of the latter, and that this requires the ability of NbHMP04 to bind copper ions. At the same time, TYLCCNV/TYLCCNB have evolved a counter-defense strategy: the βC1 protein encoded by betasatellites interacts with NbHMP04, promoting its degradation and therefore abrogating the downstream activation of defense. Based on these results, we propose the following working model: when plants are infected by viruses, NbHMP04 is upregulated and interacts with NbNPR1 to enhance its stability in a copper dose-dependent manner, thereby enhancing plant defense. Viruses, however, will deploy counter-defense strategies, exemplified here by the geminiviral βC1 protein, which interacts with NbHMP04, promotes its degradation, and enables the virus to evade plant immune response (Fig. 5). Whether the broad-spectrum antiviral effect mediated by copper ions depend on NbHMP04 and NbNPR1 remains to be investigated.

Hydroponics is an efficient plant-growing technique; in fact, hydroponic cultivation of tomatoes can be effective in increasing yields [46]. Copper, as a heavy metal element, can cause environmental pollution if inappropriately used in the field. Our results illustrate that, under hydroponic conditions, increasing the concentration of copper ions enhance plant resistance to viruses.

Heavy metal-binding proteins (HMPs) are critical in protecting plants from heavy metal toxicity and acclimating them to an ever-changing environment, while HMPs also play a significant role in plant immunity [17]. However, studies on the role of heavy metal proteins (HMPs) in plants mainly focused on their impact on plant tolerance to abiotic stresses. Few studies focus on the mechanism of how HMPs are involved in host resistance to plant viruses. In this study, we find that the NbHMP04 plays an important role in copper ions-mediated host defenses against geminivirus. Other metallic elements, such as zinc (Zn), iron (Fe), and manganese (Mn), were reported crucial for development and plant defense against viruses by regulating metabolic pathways, redox reactions, hormone signaling, and other mechanisms [47]. Therefore, more studies on the antiviral responses mediated by HMPs in plants are necessary for further research on heavy metal ion-mediated immunity.

Why does the interaction between NbHMP04 and NbNPR1 enhance the stability of the latter? NbNPR1 is degraded in salicylic acid-induced NPR1 condensates (SINCs) by the ubiquitin-proteasome (UPS) pathway [48]: NPR1 is ubiquitinated and targeted for degradation by the 26S proteasome [49]. We speculate that, through direct binding, NbHMP04 might physically cover the site of NbNPR1 ubiquitination, hence preventing its degradation.

TYLCCNB encodes βC1 to induce NbHMP04 degradation and hamper antiviral immunity. CLCuMuB βC1 activates cellular autophagy through the interaction with NbGAPCs, interfering with the NbGAPC-ATG3 association [50]. TYLCCNB βC1 can interact with the autophagic cargo protein NbNBR1, thereby escaping ubiquitination degradation by the ubiquitin ligase NbRFP1 [51]. Therefore, there seems to be a very close relationship between βC1 and plant autophagy, raising the idea that this viral protein may facilitate the binding of NbHMP04 to NbNBR1, which can be misdirected by plants into autophagosomes for degradation. In addition, the fact that some geminiviruses without satellite association, such as tomato yellow leaf curl virus (TYLCV), raises the question of how these geminiviruses escape NbHMPs-mediated plant defense in the presence of copper ions. Further efforts for elucidation of the molecular mechanism of how geminiviruses without satellite association counter HMPs-mediated defenses will broaden our horizons on the arms race between host and virus.

Soil contamination by heavy metal ions is harmful to the environment and often difficult to remediate. It is now known, however, that many heavy metal ions can promote plant resistance to pathogens. The interplay between pathogens, environmental conditions, and host plants is complex and mutually dependent. Exploring the relationship between these three aspects will undoubtedly be of great value in preventing disease outbreaks and ensuring the healthy development of ecosystems in the future.

## Data availability

The authors declare that all data supporting the findings of this study are available in the manuscript, and its Supplementary Information files are available from the corresponding author upon request.

## Declaration of competing interest

The authors declare that they have no conflicts of interest in this work.

## Appendix Supplementary materials

Image, application 1

## References

[1] DalCorso G., Manara A., Piasentin S. (2014). Nutrient metal elements in plants. Metallomics.

[2] Ghori N.-H., Ghori T., Hayat M.Q. (2019). Heavy metal stress and responses in plants. Int. J. Environ. Sci. Technol..

[3] Morkunas I., Woźniak A., Mai V.C. (2018). The role of heavy metals in plant response to biotic stress. Molecules.

[4] O'Halloran T.V., Culotta V.C. (2000). Metallochaperones, an intracellular shuttle service for metal ions. J. Biol. Chem..

[5] Culotta V.C., Klomp L.W., Strain J. (1997). The copper chaperone for superoxide dismutase. J. Biol. Chem..

[6] Glerum D.M., Shtanko A., Tzagoloff A. (1996). Characterization of COX17, a yeast gene involved in copper metabolism and assembly of cytochrome oxidase. J. Biol. Chem..

[7] Lin S.J., Culotta V.C. (1995). The *Atx1* gene of *Saccharomyces cerevisiae* encodes a small metal homeostasis factor that protects cells against reactive oxygen toxicity. Proc. Natl. Acad. Sci. U.S.A..

[8] Barth O., Zschiesche W., Siersleben S. (2004). Isolation of a novel barley cDNA encoding a nuclear protein involved in stress response and leaf senescence. Physiol. Plant.

[9] Cheng D., Tan M., Yu H. (2018). Comparative analysis of Cd-responsive maize and rice transcriptomes highlights Cd co-modulated orthologs. BMC Genom..

[10] Dykema P.E., Sipes P.R., Marie A. (1999). A new class of proteins capable of binding transition metals. Plant Mol. Biol..

[11] Rosenzweig A.C. (2002). Metallochaperones: Bind and deliver. Chem. Biol..

[12] Lu S., Wang J., Chitsaz F., Marchler-Bauer A. (2020). CDD/SPARCLE: The conserved domain database in 2020. Nucleic Acids Res..

[13] Rousselot-Pailley P., Sénèque O., Lebrun C. (2006). Model peptides based on the binding loop of the copper metallochaperone Atx1: Selectivity of the consensus sequence MxCxxC for metal ions Hg (II), Cu (I), Cd (II), Pb (II), and Zn (II). Inorg. Chem..

[14] Shoshan M.S., Tshuva E.Y. (2011). The MxCxxC class of metallochaperone proteins: Model studies. Chem. Soc. Rev..

[15] Badarau A., Dennison C. (2011). Thermodynamics of copper and zinc distribution in the cyanobacterium *Synechocystis* PCC 6803. Proc. Natl. Acad. Sci. U.S.A..

[16] Safaei R., Adams P.L., Maktabi M.H. (2012). The CXXC motifs in the metal binding domains are required for ATP7B to mediate resistance to cisplatin. J. Inorg. Biochem..

[17] Rono J.K., Sun D., Yang Z.M. (2022). Metallochaperones: A critical regulator of metal homeostasis and beyond. Gene.

[18] Wernimont A.K., Huffman D.L., Lamb A.L. (2000). Structural basis for copper transfer by the metallochaperone for the Menkes/Wilson disease proteins. Nat. Struct. Mol. Biol..

[19] Chen G., Xiong S. (2021). OsHIPP24 is a copper metallochaperone which affects rice growth. J. Plant Biol..

[20] Kim Y.-Y., Choi H., Segami S. (2009). AtHMA1 contributes to the detoxification of excess Zn (II) in Arabidopsis. Plant J..

[21] Zhang H., Zhang X., Liu J. (2020). Characterization of the Heavy-Metal-Associated Isoprenylated Plant Protein (*HIPP*) gene family from *triticeae* species. Int. J. Mol. Sci..

[22] Zhang X., Feng H., Feng C. (2015). Isolation and characterisation of cDNA encoding a wheat heavy metal-associated isoprenylated protein involved in stress responses. Plant Biol..

[23] Zschiesche W., Barth O., Daniel K. (2015). The zinc-binding nuclear protein HIPP3 acts as an upstream regulator of the salicylate-dependent plant immunity pathway and of flowering time in *Arabidopsis thaliana*. New Phytol..

[24] Radakovic Z.S., Anjam M.S., Escobar E. (2018). Arabidopsis *HIPP27* is a host susceptibility gene for the beet cyst nematode *Heterodera schachtii*. Mol. Plant Pathol..

[25] Cowan G.H., Roberts A.G., Jones S. (2018). Potato mop-top virus co-opts the stress sensor HIPP26 for long-distance movement. Plant Physiol..

[26] Zhou P., Zavaliev R., Xiang Y. (2023). Seeing is believing: Understanding functions of NPR1 and its paralogs in plant immunity through cellular and structural analyses. Curr. Opin. Plant Biol..

[27] Kumar S., Zavaliev R., Wu Q. (2022). Structural basis of NPR1 in activating plant immunity. Nature.

[28] Zhang Y., Fan W., Kinkema M. (1999). Interaction of NPR1 with basic leucine zipper protein transcription factors that bind sequences required for salicylic acid induction of the *PR-1* gene. Proc. Natl. Acad. Sci. U.S.A..

[29] Potlakayala S.D., DeLong C., Sharpe A. (2007). Conservation of non-expressor of pathogenesis-related genes1 function between *Arabidopsis thaliana* and *Brassica napus*. Physiol. Mol. Plant Pathol..

[30] Verma R., Hamsa S., Mandal S. (2023). Expression of *Arabidopsis NPR1* (*AtNPR1*) in *Brassica juncea var Varuna* confers significant resistance against *Sclerotinia sclerotiorum* and *Alternaria brassicae*. Physiol. Mol. Plant Pathol..

[31] Liu Y., Schiff M., Marathe R. (2002). Tobacco *Rar1, EDS1* and *NPR1/NIM1* like genes are required for *N*-mediated resistance to tobacco mosaic virus. Plant J..

[32] Li F., Qiao R., Wang Z. (2022). Occurrence and distribution of geminiviruses in China. Sci. China Life Sci..

[33] Brown J.K., Zerbini F.M., Navas-Castillo J. (2015). Revision of *Begomovirus* taxonomy based on pairwise sequence comparisons. Arch. Virol..

[34] Harrison B.D., Robinson D.J. (1999). Natural genomic and antigenic variation in whitefly-transmitted geminiviruses (begomoviruses). Annu. Rev. Phytopathol..

[35] Li F., Yang X., Bisaro D.M. (2018). The βC1 protein of geminivirus-betasatellite complexes: A target and repressor of host defenses. Mol. Plant.

[36] Gong P., Tan H., Zhao S. (2021). Geminiviruses encode additional small proteins with specific subcellular localizations and virulence function. Nat. Commun..

[37] Yang J.-Y., Iwasaki M., Machida C. (2008). βC1, the pathogenicity factor of TYLCCNV, interacts with AS1 to alter leaf development and suppress selective jasmonic acid responses. Genes Dev..

[38] Yang X., Xie Y., Raja P. (2011). Suppression of methylation-mediated transcriptional gene silencing by βC1-SAHH protein interaction during geminivirus-betasatellite infection. PLoS Pathog..

[39] Jia Q., Liu N., Xie K. (2016). CLCuMuB βC1 subverts ubiquitination by interacting with NbSKP1s to enhance geminivirus infection in *Nicotiana benthamiana*. PLoS Pathog..

[40] Zhou X., Xie Y., Tao X. (2003). Characterization of DNAbeta associated with begomoviruses in China and evidence for co-evolution with their cognate viral DNA-A. J. Gen. Virol.

[41] Xin Z., Chen J. (2012). A high throughput DNA extraction method with high yield and quality. Plant Methods.

[42] Jiang Z., Yang M., Cao Q., Wang A., Li Y. (2022). Plant Virology: Methods and Protocols.

[43] Tehseen M., Cairns N., Sherson S. (2010). Metallochaperone-like genes in *Arabidopsis thaliana*. Metallomics.

[44] Yao S., Kang J., Guo G. (2022). The key micronutrient copper orchestrates broad-spectrum virus resistance in rice. Sci. Adv..

[45] Wu Y., Zhang D., Chu J.Y. (2012). The Arabidopsis NPR1 protein is a receptor for the plant defense hormone salicylic acid. Cell Rep..

[46] Fayezizadeh M.R., Ansari N.A.Z., Albaji M. (2021). Effects of hydroponic systems on yield, water productivity and stomatal gas exchange of greenhouse tomato cultivars. Agric. Water Manag..

[47] Rai S., Singh P., Mankotia S. (2021). Iron homeostasis in plants and its crosstalk with copper, zinc, and manganese. Plant Stress.

[48] Zavaliev R., Mohan R., Chen T. (2020). Formation of NPR1 condensates promotes cell survival during the plant immune response. Cell.

[49] Bashore C., Prakash S., Johnson M.C. (2023). Targeted degradation via direct 26S proteasome recruitment. Nat. Chem. Biol..

[50] Ismayil A., Yang M., Haxim Y. (2020). Cotton leaf curl Multan virus betaC1 protein induces autophagy by disrupting the interaction of autophagy-related protein 3 with glyceraldehyde-3-phosphate dehydrogenases. Plant Cell.

[51] Zhou T., Zhang M., Gong P. (2021). Selective autophagic receptor NbNBR1 prevents NbRFP1-mediated UPS-dependent degradation of βC1 to promote geminivirus infection. PLoS Pathog..

