# Supplementary material for: A key heavy metal-binding protein orchestrates plant resistance against a geminivirus
Source: Fundam Res. 2024 Dec 27;6(2):847–55. doi: 10.1016/j.fmre.2024.12.005 (PMC13069656; doi:10.1016/j.fmre.2024.12.005)
Supplement: Supplementary file 1 [file mmc1.pdf]

Supplementary Information

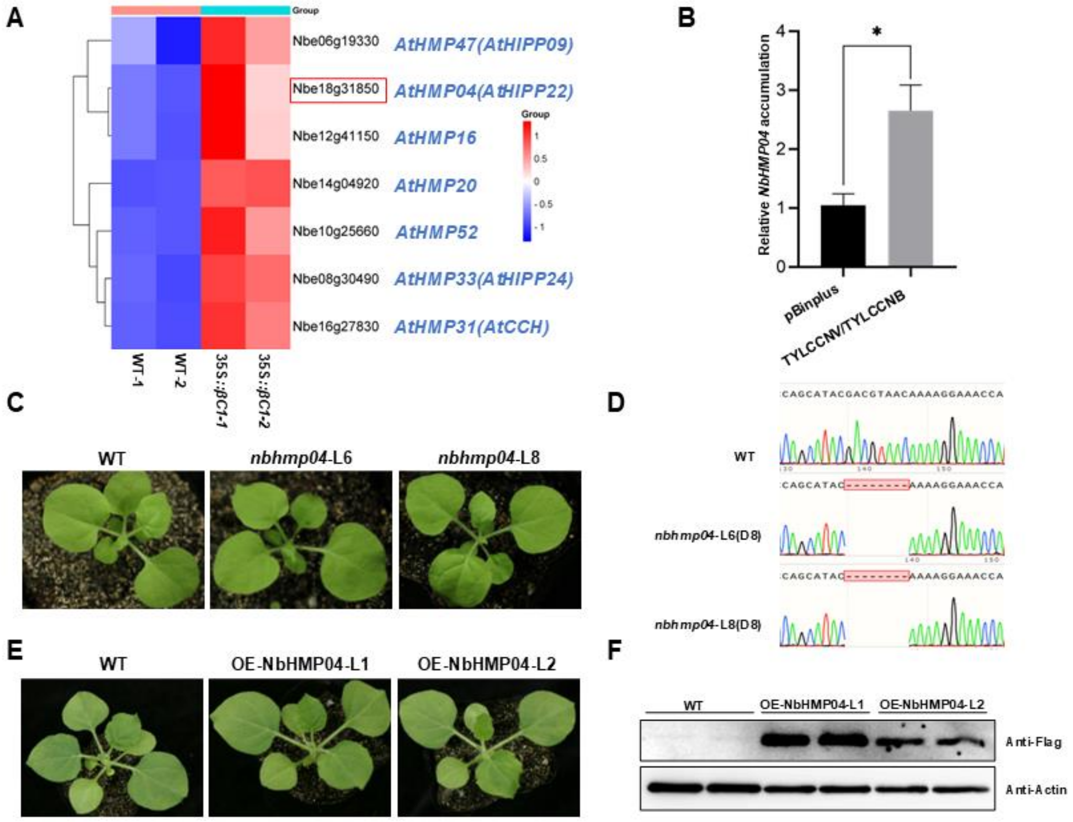

Figure S1. Generation of *NbHMP04* knock-out and overexpressing plants. **(A)** Expression heatmap of differentially expressed genes of wild-type or *35S::βC1 N. benthamiana* plants, showing selected heavy metal protein genes that were induced more prominently in *35S::βC1 N. benthamiana* plants in comparison to wild-type *N. benthamiana* plants. The bright blue text on the right of figure is the name of the corresponding homologous gene in *Arabidopsis thaliana*. The blue scale indicates a low level of transcript abundance and the red scale indicates a high level of transcript abundance. The red box is the object gene of this paper. **(B)** Quantitative real-time PCR (qPCR) showing accumulation of *NbHMP04* in TYLCCNV/TYLCCNB infectious clone or pBinplus (as negative control) systemically infected leaves of *N. benthamiana* plants. Data are presented as means ± SD of three biological replicates. Statistical analyses were performed using the Student's t-test. *, $p<0.05$. *NbActin* was used as the internal reference gene. **(C)** Phenotype of

four-week-old wild-type， *nbhmp04*-L6, and *nbhmp04*-L8 *N. benthamiana* plants. **(D)** DNA sequencing and sequence alignment results confirmed an 8-base deletion in the *NbHMP04* gene sequence. These nucleotide deletions cause frame shifts in the coding region of *NbHMP04*. The transgene T1 plant *nbhmp04*-L6 and *nbhmp04*-L8 *N. benthamiana* plants were used for sequencing analyses. D8 represents 8-base deletion. **(E)** Phenotype of four-week-old wild-type, OE-NbHMP04-L1, or OE-NbHMP04-L2 *N. benthamiana* plants. **(F)** Western blot showing protein accumulation of NbHMP04-Flag in wild-type, OE-NbHMP04-L1, or OE-NbHMP04-L2 *N. benthamiana* plants. NbActin acts as the loading control.

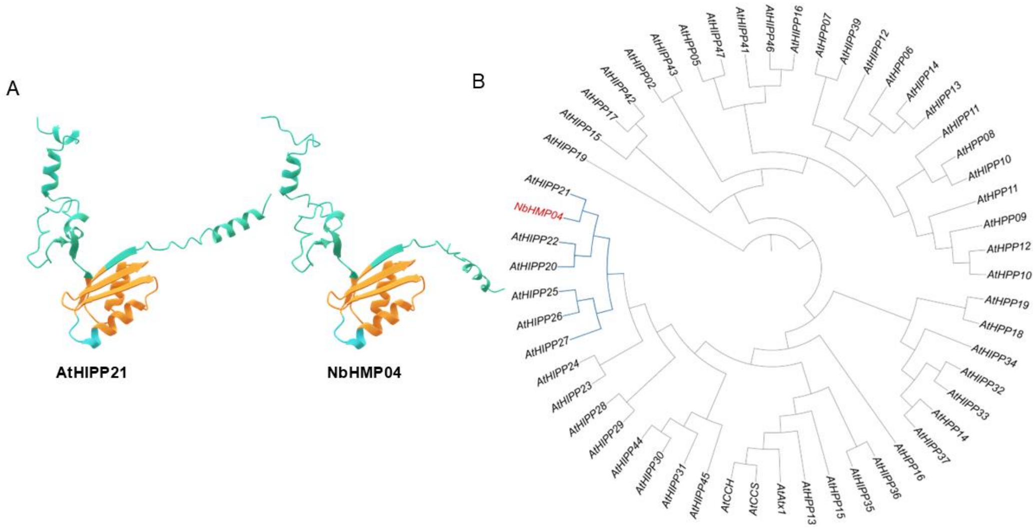

Figure S2. Protein structure prediction and phylogenetic tree. **(A)** Protein structure prediction by AlphaFold II. AtHIPP21 was downloaded from UniProt. NbHMP04 was analyzed by AlphaFold II, then viewed using RCSB Pub view. **(B)** Construction of a phylogenetic tree of NbHMP04 and orthologs in *Arabidopsis thaliana*. The evolutionary tree was built using MAGE's Neighbor-Joining method and utilizing the online tool iTOL for beautification.

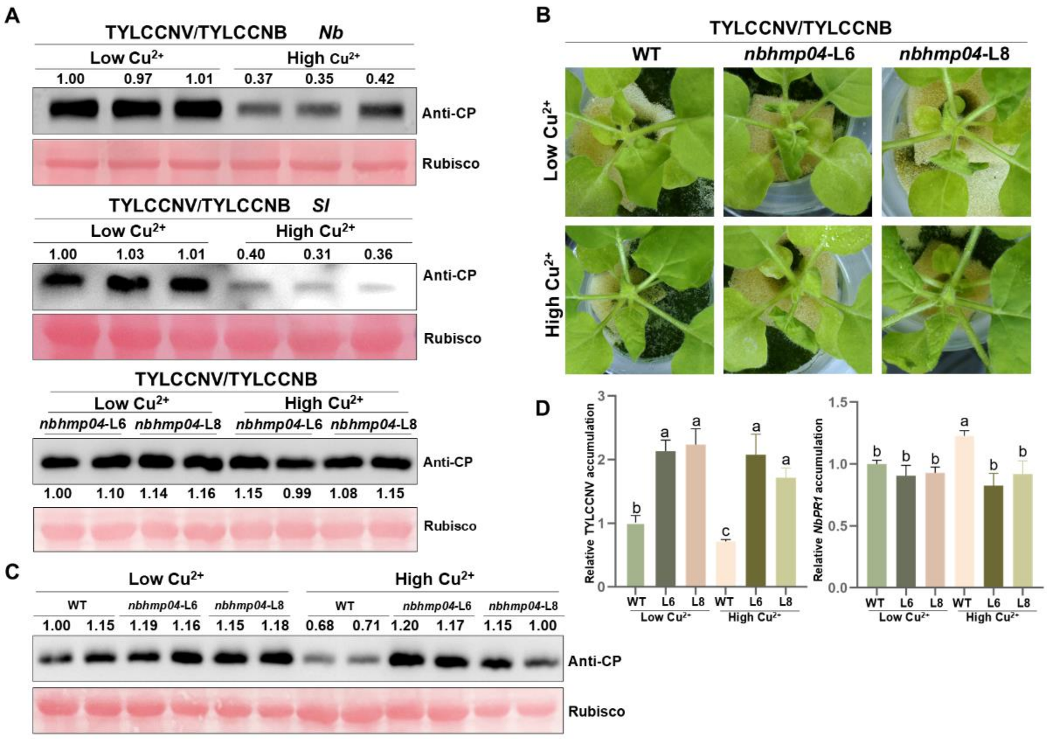

Figure S3. The role of NbHMP04 in plant defense requires its ability to bind copper ions. **(A)** Western blot showing protein accumulation of TYLCCNV CP in systemically infected leaves of wild-type *N. benthamiana* plants (upper panel), *S. lycopersicum* (middle panel) or wild-type, *nbhmp04*-L6, and *nbhmp04*-L8 *N. benthamiana* plants (lower panel). These were grown in low Cu^{2+} (0.3 μM Cu^{2+} hydroponic solution) or high Cu^{2+} (3 μM Cu^{2+} hydroponic solution) infected with TYLCCNV/TYLCCNB infectious clones. The corresponding Ponceau S staining of the large Rubisco subunit acts as the loading control. **(B)** Symptoms of wild-type, *nbhmp04*-L6 and *nbhmp04*-L8 *N. benthamiana* plants grown in low Cu^{2+} or high Cu^{2+} hydroponic solution and infected with TYLCCNV/TYLCCNB infectious clones at 6 days post-inoculation (dpi). **(C)** Western blot showing protein accumulation of TYLCCNV CP in systemically infected leaves of wild-type, *nbhmp04*-L6 and *nbhmp04*-L8 *N. benthamiana* from (B). The corresponding Ponceau S staining of the large

Rubisco subunit acts as the loading control. **(D)** Quantitative real-time PCR showing accumulation of *TYLCCNV CP* (left panel) and *NbPR1* (right panel) from (B) systemically infected leaves. Data are presented as means ± SD of three biological replicates. The letters a, b and c showing statistically significant differences between variables. *NbActin* was used as the internal reference gene.

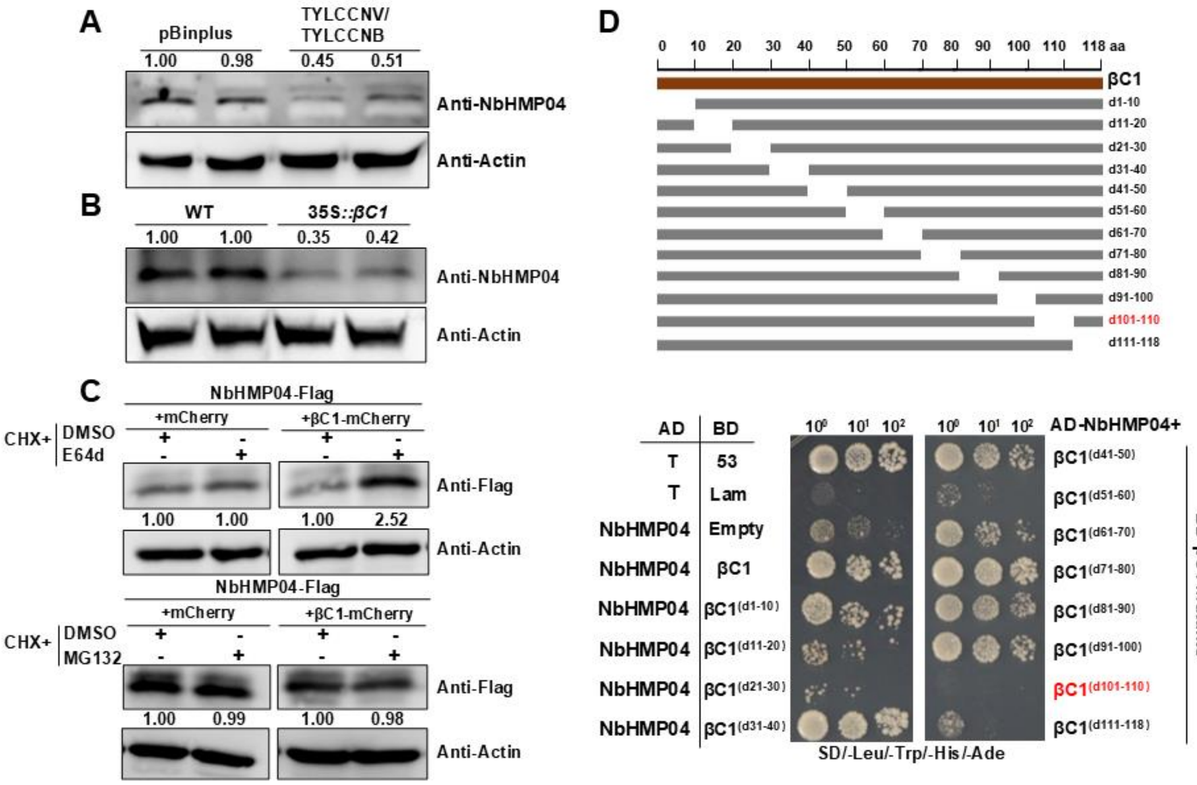

Figure S4. Accumulation of NbHMP04 is decreased due to the interaction with βC1. **(A)** Expression of endogenous NbHMP04 protein in *N. benthamiana* plants after inoculation with TYLCCNV/TYLCCNB infectious clone and pBinplus (as a negative control). NbActin acts as the loading control. **(B)** Accumulation of endogenous NbHMP04 protein in wild-type and *35S::βC1 N. benthamiana* plants. NbActin acts as the loading control. **(C)** In *vivo* degradation inhibition analysis of NbHMP04 using 100 μM MG132, 50 μM E64d and 0.1 % DMSO respectively. A construct to express NbHMP04-Flag was co-transformed with constructs to express either mCherry (as the

negative control) or βC1-mCherry in *N. benthamiana* leaves. 100 μM CHX used to inhibit the synthesis of new protein. 100 μM MG132 was used to inhibit degradation by the 26S proteasome, 50 μM E64d was used to inhibit degradation by autophagy and 0.1 % DMSO as a control. NbActin acts as the loading control. **(D)** Interaction between NbHMP04 and βC1 truncated mutants in yeast. We generated a mutant form of βC1 by truncating it at every 10 amino acids, as shown in the upper figure, The d1-10 variant was generated by truncating the first 10 amino acids, thereby retaining the βC1 mutant sequence spanning from the 11th to the 118th amino acids. Subsequently, a potential interaction between these truncated forms of βC1 and NbHMP04 was assessed in yeast as shown in the lower of figure S4D.

Table S1. Primers used in this study (5'–3')

Primers	Sequences
SgRNA sequence-1	CCAGCATACGACGTAACAAAAGG
SgRNA sequence-2	GAGTCAAGAATGCCGTTAAACGG
pCambia1300-NbHMP04-Flag-F	GTACCCGGGGATCCATGGGTGTTCTTGATTATTTTTC
pCambia1300-NbHMP04-Flag-R	CCTTATAGTCGTCGACTCTAGACATAATAGAGCAAGCATTG
q-TYLCCNV-F	AGAAGACAAATGTGGTCCAACAGG
q-TYLCCNV-R	GCAATTAAAGACTTGTTGGAAATCCAT
q-NbActin2-F	TGGTCGTACCACCGGTATTGTGTT
q-NbActin2-R	TCACTTGCCCATCAGGAAGCTCAT
Cas9-det-NbHMP04-F	GAACCAACTTAACTTCTTGCC
Cas9-det-NbHMP04-R	GTTATGAATCTTCTTCACTTG
pGBKT7-NbHMP04-F	CATGGAGGCCGAATTCCCATGGGTGTTCTTGATTATTTTTC
pGBKT7-NbHMP04-R	GGTCGACGGATCCCCTTACATAATAGAGCAAGCATTGG
pGADT7-NbHMP04-F	GAGGCCAGTGAATTCCACATGGGTGTTCTTGATTATTTTTCAAAC
pGADT7-NbHMP04-R	CGAGCTCGATGGATCCCGTTTACATAATAGAGCAAGCATTGG
q-NbHMP04-F	GTTCTTGATTATTTTTCAAACTTTTGC
q-NbHMP04-R	CTATTCAACACTCTCTGTGGTTC
nYFP-NbHMP04-F	CGATAGTTAATTAAATGGGTGTTCTTGATTATTTTTC
nYFP-NbHMP04-R	CTCCTCCACTAGTTTACATAATAGAGCAAGCATTG
NbHMP04-GFP-F	GGTACCCGGGGATCCATGGGTGTTCTTGATTATTTTTC
NbHMP04-GFP-R	CACCATGTCGACTCTAGACATAATAGAGCAAGC
NbHMP04^{mHMA}-F	GATGGATGGTGATGGAGGCGAAAGAAGAGTCAAGAATGC

NbHMP04^{mHMA}-R	CTCTTCTTTCGCCTCCATCACCATCCATCTTCACTTTTATCTCCAC
cYFP-NbHMP04-F	CGATAGTTAATTAAATGGGTGTTCTTGATTATTTTTC
cYFP-NbHMP04-R	TGCCACTCCTCCACTAGTCATAATAGAGCAAGCATTG
pGBKT7-βC1-F	GCCATGGAGGCCGAATTCATGACTATCAAATACAAC
pGBKT7-βC1-R	CAGGTCGACGGATCCCCGGTTATACATCTGAATTTGTAAATAC
pGBKT7-βC1(V101A)-F	GAAGAAGCCCCAGCAGAAGATATAGATGTAGGGGATG
pGBKT7-βC1(V101A)-R	CTATATCTTCTGCTGGGGCTTCTTCCATCATGATTATG
pGBKT7-βC1(D103A)-F	GCCCCAGTAGAAGCTATAGATGTAGGGGATGAG
pGBKT7-βC1(D103A)-R	CATCTATAGCTTCTACTGGGGCTTCTTCCATC
pGBKT7-βC1(I104A)-F	GTAGAAGATGCAGATGTAGGGGATGAGTATGATG
pGBKT7-βC1(I104A)-R	CTACATCTGCATCTTCTACTGGGGCTTCTTC
pGBKT7-βC1(V106A)-F	GAAGATATAGATGCAGGGGATGAGTATGATG
pGBKT7-βC1(V106A)-R	CCCCTGCATCTATATCTTCTACTGGGGCTTC
pGBKT7-βC1(D108A)-F	GATGTAGGGGCTGAGTATGATGTATTTAC
pGBKT7-βC1(D108A)-R	CATACTCAGCCCCTACATCTATATCTTCTAC
pGBKT7-βC1(E109A)-F	GTAGGGGATGCGTATGATGTATTTAC
pGBKT7-βC1(E109A)-R	CATCATACGCATCCCCTACATCTATATCTTC
pGBKT7-βC1(Y110A)-F	GGGATGAGGCTGATGTATTTACAAATT
pGBKT7-βC1(Y110A)-R	GTAAATACATCAGCCTCATCCCCTACATCTATATC
βC1-mCherry-F	GGACGAGCTCGGTACCATGACTATCAAATACAACAACATGAAGG
βC1-mCherry-R	GTCGACTCTAGAGGATCCTACATCTGAATTTGTAAATACATC
cYFP-βC1-F	GAACGATAGTTAATTAAATGACTATCAAATACAACAACATGAAGG
cYFP-βC1-R	TGCCACTCCTCCACTAGTTACATCTGAATTTGTAAATACATC
nYFP-βC1-F	GAACGATAGTTAATTAAATGACTATCAAATACAACAACATGAAGG
nYFP-βC1-R	GCCACCTCCTCCACTAGTTACATCTGAATTTGTAAATACATC
pYES2-NbHMP04-F	GAATATTAAGCTTGGTACCGAGATGGGTGTTCTTGATTATTTTTC
pYES2-NbHMP04-R	CCGTTACTAGTGGATCCGACATAATAGAGCAAGC
βC1^{Y110A}-3HA-F	GGATCCTGGCTCGAGATGACTATCAAATACAACAACATG
βC1^{Y110A}-3HA-R	GAACATCGTATGGGTAGAGCTCTACATCTGAATTTGTAAATAC
pGBKT7-NbNPR1-F	CATGGAGGCCGAATTCATGGATAATAGTAGGACTGCGTTTTC
pGBKT7-NbNPR1-R	CGACGGATCCCCGGCTATTTCCTAAAAGGGAGC
Flag-Gus-F	GGTACCCGGGGATCCATGTTACGTCCTGTAG
Flag-Gus-R	CTGCAGGTCGACTCTAGACGCGTGGTTACAGTCTTGC
NbNPR1-GFP-F	GGTACCCGGGGATCCATGGATAATAGTAGGAC
NbNPR1-GFP-R	GCTCACCATGTCGACTCTAGATTTCCTAAAAGGGAGC
NbNPR1-3XFlag-F	GAGCTCGGTACCCGGGGATCCATGGATAATAGTG
NbNPR1-3XFlag-R	GTCCTTATAGTCGTCGACTCTAGATTTCCTAAAAGGGAG
nYFP-NPR1-F	CGAACGATAGTTAATTAAATGGATAATAGTGGGACTGCGTTTTC
nYFP-NPR1-R	GCCACCTCCTCCACTAGTTTTCCTAAAAGGGAGCTTATTG
NbHMP04-mcherry-F	CGGGGGACGAGCTCGGTACCATGGGTGTTCTTG
NbHMP04-mcherry-R	CATGTCGACTCTAGAGGATCCCATAATAGAGCAAGC
